# Familial Renal Cancer: Molecular Genetics and Surgical Management

**DOI:** 10.1155/2011/658767

**Published:** 2011-08-22

**Authors:** Glen W. Barrisford, Eric A. Singer, Inger L. Rosner, W. Marston Linehan, Gennady Bratslavsky

**Affiliations:** ^1^Department of Urology, National Naval Medical Center, Bethesda, MD 20889, USA; ^2^Urologic Oncology Branch, Center for Cancer Research, National Cancer Institute, National Institutes of Health, 10 Center Drive, Building 10, Room 1-5940, Bethesda, MD 20892, USA; ^3^Department of Urology, Upstate Medical University, SUNY, Syracuse, NY 13210, USA

## Abstract

Familial renal cancer (FRC) is a heterogeneous disorder comprised of a variety of subtypes. Each subtype is known to have unique histologic features, genetic alterations, and response to therapy. Through the study of families affected by hereditary forms of kidney cancer, insights into the genetic basis of this disease have been identified. This has resulted in the elucidation of a number of kidney cancer gene pathways. Study of these pathways has led to the development of novel targeted molecular treatments for patients affected by systemic disease. As a result, the treatments for families affected by von Hippel-Lindau (VHL), hereditary papillary renal carcinoma (HPRC), hereditary leiomyomatosis renal cell carcinoma (HLRCC), and Birt-Hogg-Dubé (BHD) are rapidly changing. We review the genetics and contemporary surgical management of familial forms of kidney cancer.

## 1. Introduction

Renal cell carcinoma (RCC) has a global impact with approximately 111,100 new cases and 43,000 deaths from the disease among men in developed countries in 2008 alone [1]. In 2010, RCC ranked as the seventh and eighth most common malignancy in men and women in the United States, respectively, and 58,240 new cases and 13,040 deaths were expected [2]. Americans face a diagnosis of renal malignancy at a rate of approximately 1 in 67 over the course of their lifetime [3]. The increased availability and use of cross-sectional imaging as well as other imaging modalities tends to diagnose renal tumors at earlier stages and often as incidental findings. However, despite the increased and advanced detection, the associated mortality rate has not declined [4]. Unexpectedly, the increased incidence of RCC cannot be entirely explained by the more widespread use of imaging modalities [5].

**Figure 1:**
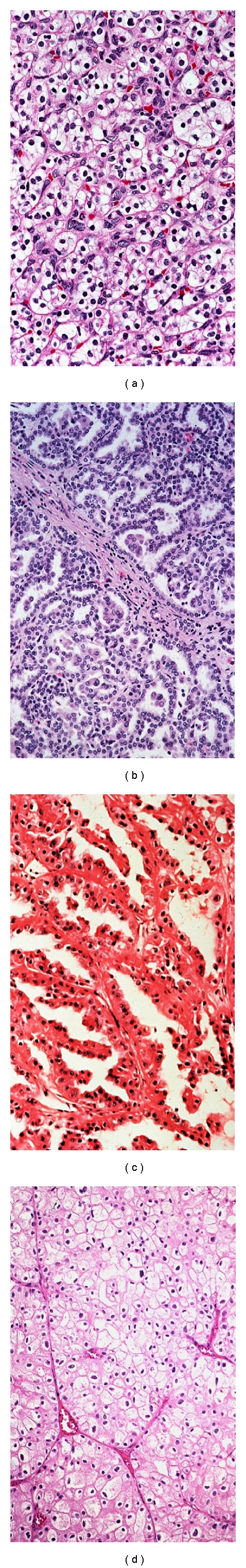
Histopathology of the most common malignant renal neoplasms. (a) Clear cell; (b) papillary type 1; (c) papillary type 2; and (d) chromophobe. (From Linehan et al. [10], with permission.)

Surgical resection has historically been the mainstay of therapy as RCC is known to be resistant to radiation and traditional chemotherapy [6, 7]. Although surgical resection is often curative, up to 30% of patients will present with systemic disease, while an additional 30% will develop metastatic lesions in followup after an initial presentation of organ confined disease [8]. Treatment of systemic disease has often been challenging due to the fact that RCC represents a heterogeneous spectrum of diverse entities. Each subtype of renal malignancy is known to possess unique clinical characteristics, genetic alterations, and has varied responses to therapy. The dominant malignant subtypes recognized by the Heidelberg classification system include clear cell (conventional) (70–80%), papillary (chromophile) (10–15%), chromophobe (3–5%), and collecting duct (1%) tumors [9]. Papillary tumors are further stratified into type 1 (5%) and type 2 (10%) based upon genetic and histologic variation [10–12] (Figure 1).

RCC can exist as both hereditary and sporadic entities. Sporadic RCC typically presents as a solitary lesion and will occur more commonly in patients in the sixth decade and beyond. Conversely, hereditary forms of kidney cancer often present with multifocal, bilateral tumors and may present in far younger patients [13]. Many cases of hereditary renal malignancy go unrecognized or unreported as this spectrum of diseases is not well understood [14]. Moderate estimates place hereditary disease forms at 3–5% of the overall number of diagnoses [15, 16]. Liberally defined, familial renal cancer (FRC) is noted to exist when more than one member of a family presents with a single malignancy or collection of tumors [16]. 

The identification of FRC is critical in that it allows for the early screening of families, vigilant followup for those affected, appropriate and measured interventions when needed, and the reduction of disease-related morbidity and mortality. When a patient's family history is positive for kidney cancer, or a patient with one or more of the physical/radiographic findings outlined in Table 1 is found to have a renal mass, further investigation and referral to a genetic counselor are often reasonable. The American Society of Clinical Oncology's recently published guidelines on genetic testing for cancer susceptibility concisely identify and explain many of the ethical issues associated with germ line analysis [17].

FRC is a heterogeneous disease comprised of a spectrum of varied histologic subtypes. The fundamental link among the forms of FRC with a known genetic alteration is that they are metabolic in nature, as the genes involved with FRC are associated with abnormalities in oxygen, iron, glucose, and energy sensing [18]. The identification of genetic aberrations associated with familial forms of kidney cancer has led to the elucidation of a number of interrelated metabolic pathways. As a result, a number of targeted molecular therapies have developed over the past half decade [19]. This has altered the management of advanced and systemic RCC and provided additional approaches to cytokine-based therapies for the systemic therapy of clear cell tumors [20, 21]. The treatment of choice for nonclear cell histologies is presently less well defined [22]. Understanding the genetic abnormalities and the pathways leading to the tumorigenesis of FRC provided the opportunity for the development of novel forms of therapies targeting these cancer gene pathways (Table 1).

## 2. Von Hippel-Lindau

Von Hippel-Lindau (VHL) is a hereditary renal cancer syndrome associated with clear cell renal tumors. The inheritance pattern is autosomal dominant, and the incidence is 1 in 36,000 live births [10, 24, 25]. The disease can manifest clinically with renal tumors, adrenal pheochromocytomas, retinal angiomas, central nervous system hemangioblastomas, pancreatic cysts, neuroendocrine tumors, endolymphatic sac tumors, and cystadenomas of the epididymis and broad ligament. Each of these tumors is known to be highly vascular. Improvement in treatment for central nervous system tumors has had the effect of elevating metastatic renal cell carcinoma to the leading cause of mortality among these patients. Renal tumors appear in 35–45% of affected individuals, can be solid or cystic, and are of clear cell histology [26]. Patients can develop up to 600 microscopic tumors and over 1100 cysts per kidney [13, 24].

VHL-associated tumors were noted to have a consistent loss of the short arm of chromosome 3 [27]. Genetic linkage studies in patients with hereditary and sporadic renal cancer led to the identification of the *VHL* gene on the short arm of chromosome 3 (3p26-25) [28, 29]. This gene is a tumor suppressor, and the loss of a single normal allele was observed in the VHL gene in kidney cancer tissue samples [30]. This observation suggested that there existed an inherited gene at this location. This germline alteration was observed in VHL-associated kidney cancer and is found in nearly 100% of VHL families [31]. Somatic VHL alterations are observed in a high percentage of sporadic clear cell renal tumors but are absent in papillary, chromophobe, or collecting duct tumors [32]. There are currently more than 300 known alterations available for testing that involve the VHL gene [33, 34]. The location and type of the *VHL* gene alteration can result in the expression of different phenotypes. Penetrance is widely varied, and certain traits (pheochromocytomas) tend to be clustered in certain families while absent in others [35]. Patients with partial germline deletions are noted to have a higher incidence of RCC and greater extent of involvement among various organ systems as compared to those with complete deletions [33, 36, 37].

**Figure 2:**
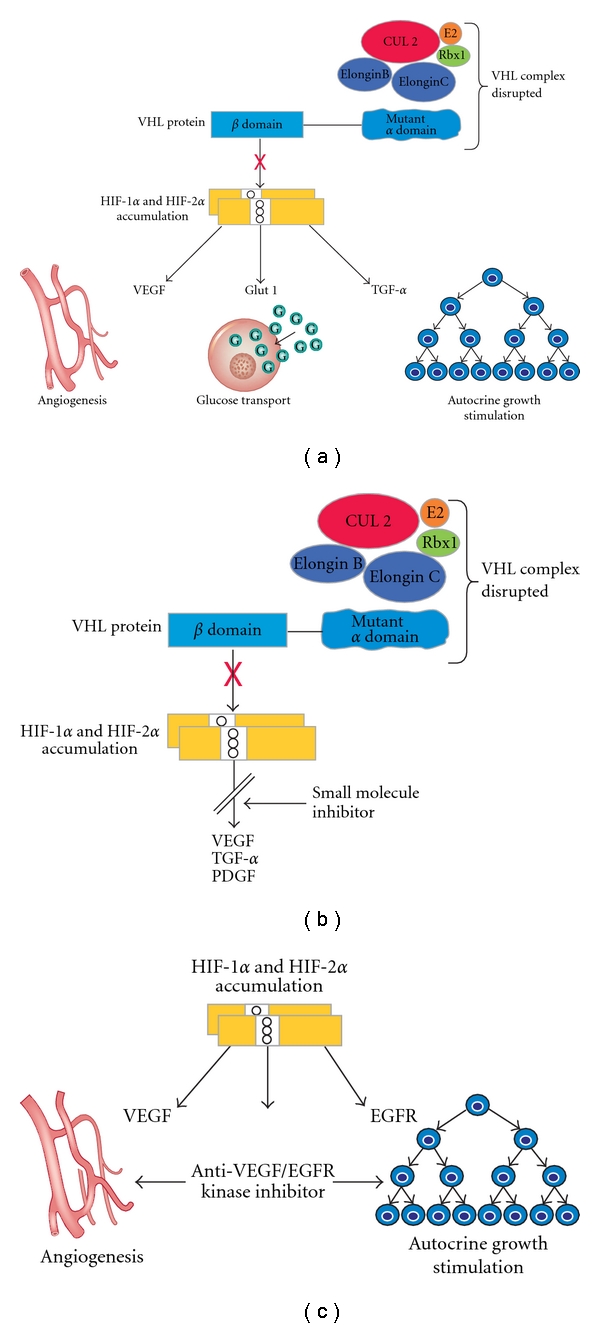
The VHL complex targets HIF-1*α* and HIF-2*α* for ubiquitin-mediated degradation. In clear cell RCC, an alteration in the VHL gene in the *α* or *β* domain disrupts HIF degradation. HIF overaccumulates leading to increased transcription of downstream genes. (a) VHL alteration; (b) VHL/HIF pathway molecular targeting; and (c) VHL/HIF downstream molecular targeting. (From Linehan and Zbar [23], with permission.)

VHL gene function has been rigorously studied. It is a relatively small gene encoding 854 nucleotides on three exons and encodes the VHL protein [29]. The VHL protein (pVHL) forms a complex with elongin B, elongin C, Cul 2, and Rbx1 [38–40]. This complex targets the hypoxia-inducible factors (HIF), HIF-1*α* and HIF-2*α*, and is essential for ubiquitin-mediated degradation [41, 42]. The transcription products of HIF-1*α* and HIF-2*α* are known to regulate a number of downstream genes that are involved in tumorigenesis. The main examples of these genes are vascular endothelial growth factor (VEGF), platelet derived growth factor (PDGF), epidermal growth factor receptor (EGFR), transforming growth factor (TGF-*α*), and glucose transporter (GLUT-1). During conditions of normal tissue oxygen levels, the VHL complex binds to HIF initiating ubiquitin-mediated degradation. However, during conditions of low tissue oxygen levels, the VHL complex does not degrade HIF which results in a surge of HIF levels and an upregulation of transcription of HIF-dependent genes [13, 43]. In the instance of clear cell RCC, a *VHL* gene alteration changes the alpha domain (which binds elongin C/B and Cul 2) or the beta subunit (which targets HIF for breakdown). These changes result in a buildup of HIF and consequently an increased expression of the downstream genes (Figure 2). The pVHL protein causes an effect similar to hypoxia and can activate pathways for cellular proliferation and neovascularization. This effect takes place under normoxic conditions and has been termed “pseudohypoxia” [12, 44]. Many of the current therapeutic management approaches for clear cell RCC are based upon the targeting of the receptors for HIF-regulated genes [45]. The agents currently approved by the US Food and Drug Administration to treat metastatic clear cell RCC via the targeting of VHL transcription products include sunitinib, sorafenib, bevacizumab plus interferon-*α*, pazopanib, temsirolimus, and everolimus [19]. These agents form the foundation for systemic therapy in a disease that has been resilient in the face of cytotoxic chemotherapy and radiotherapy [6, 7, 21]. Despite the great progress in the arena of targeted therapy, the bulk of the work has been in clear cell histology, and treatment for other histologic subtypes is presently less well established [22].

## 3. Hereditary Papillary Renal Carcinoma

Hereditary papillary renal carcinoma (HPRC) is an inherited renal cancer syndrome in which affected individuals are at risk of developing multifocal bilateral type 1 papillary renal carcinoma. It follows an autosomal dominant inheritance pattern with very high penetrance, meaning that there is a high likelihood of a person developing papillary RCC by age 80 [46]. Affected individuals are generally at risk of developing tumors in the sixth through eighth decades [47]. The only involved organ in HPRC is the kidney. The tumors are typically well differentiated but are malignant and can metastasize. Initial descriptions of the disease describe it as “late onset”, occurring in the later decades of life [46, 48]. However, within the last decade, an early onset form of HPRC has been identified [49]. Renal tumors in these patients are often diagnosed incidentally [50]. 

Computed tomography (CT) and magnetic resonance imaging (MRI) are the imaging modalities of choice when evaluating patients with HPRC as they demonstrate greater sensitivity when compared to renal ultrasound [51]. However, these lesions are typically small and hypovascular with poor enhancement on CT imaging. HPRC lesions can easily be mistaken for renal cysts, and a high index of suspicion is needed. In these cases, ultrasound is a useful adjunct to differentiate cystic from solid lesions, although the solid lesions may be isoechoic to normal renal parenchyma.

**Figure 3:**
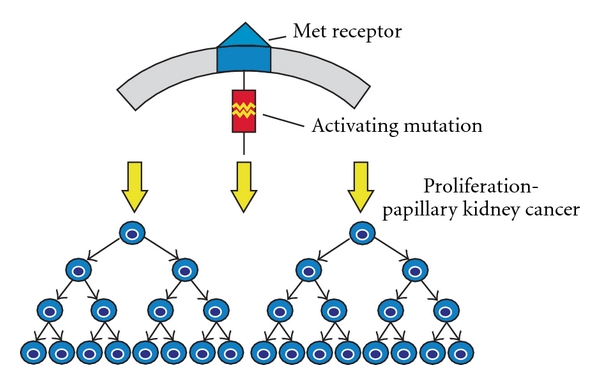
Alterations in the intracellular tyrosine kinase domain of the *MET* proto-oncogene are found in patients with HPRC. These mutations result in the activation of the MET pathway. (From Linehan et al. [10], with permission.)

HPRC-affected families were evaluated and found to be devoid of abnormalities on chromosome 3. However, within the first few years of the identification of the syndrome, research yielded the gene responsible for HPRC on chromosome 7q31 [46, 48]. The germline alteration in HPRC activates a proto-oncogene. The missense mutations in the tyrosine kinase domain of the *MET* proto-oncogene at 7q31 are responsible for the constitutive activation of the MET protein. The MET transmembrane protein is located at a hepatocyte growth factor receptor site, and a tyrosine kinase domain is located intracellularly [52]. Hepatocyte growth factor activates MET tyrosine phosphorylation which in turn induces proliferation and differentiation of epithelial and endothelial cells, cell branching, and invasion [13, 53] (Figure 3). *MET* alterations in somatic cells have been identified in a division of patients with sporadic papillary type 1 renal cancer [54]. Changes in the *MET* gene involve ligand-independent activation of the intracytoplasmic tyrosine kinase domain leading to activation of the hepatocyte growth factor (HGF)/MET pathway resulting in tumor formation [55, 56]. HPRC families retain germline changes in the *MET* gene with nonrandom duplication of chromosome 7 with the altered *MET* allele [57].

Molecular targeting aimed to inhibit HGF, and the subsequent downstream pathways could be a potential therapy of papillary type 1 RCC in patients with HPRC [12]. For example, foretinib is an oral tyrosine kinase receptor inhibitor that targets c-MET and VEGFR2 that has been studied in a phase II multicenter trial [58].

## 4. Hereditary Leiomyomatosis and Renal Cell Cancer

Hereditary leiomyomatosis and renal cell cancer (HLRCC) is a hereditary renal cancer syndrome that was initially reported in 2001 by Launonen et al. [60]. Individuals affected with this syndrome are at risk of developing papillary type 2 renal tumors as well as cutaneous and uterine leiomyomas. These aggressive renal lesions can be mistaken for collecting duct RCC tumors [61]. Although papillary type 2 lesions can occur in a sporadic fashion, those associated with HLRCC tend to occur as unilateral solitary lesions that are very aggressive, prone to metastasis, and lethal if afforded the opportunity to progress [62, 63]. The gene responsible for HLRCC was identified by Tomlinson et al. on chromosome 1 (1q42-44) and is known as fumarate hydratase (*FH*). This gene functions as a tumor suppressor, and both alleles are inactivated in tumor tissue [64]. The *FH* gene is inherited in an autosomal dominant fashion with high penetrance.

**Figure 4:**
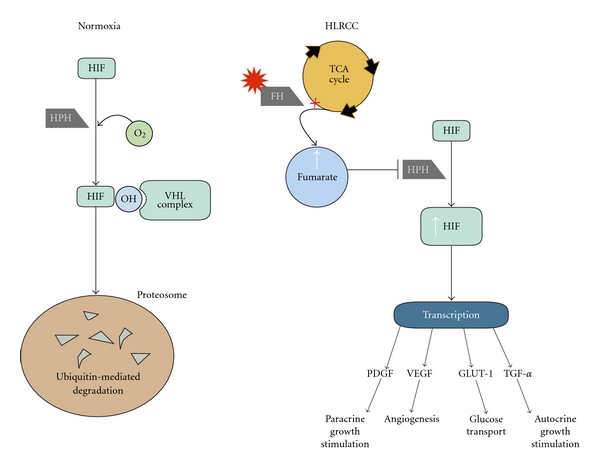
In a normoxic environment, HIF is hydroxylated by HPH allowing the VHL complex to initiate ubiquitin-mediated breakdown in the proteosome. In HLRCC, *FH* alteration results in a buildup of fumarate. Fumarate competitively inhibits HPH allowing a rise in HIF levels and subsequent transcription of downstream genes. (From Pfaffenroth and Linehan [59], with permission.)

Fumarate hydratase is a critical enzyme in aerobic metabolism. Its role in the Krebs cycle is the conversion of fumarate to malate. Alteration to FH upregulates HIF and creates a pseudohypoxic environment that is similarly seen in VHL. When FH is inactivated, fumarate levels build up and competitively inhibit HIF prolyl hydroxylase (HPH). HPH is a key enzymatic regulator of intracellular HIF levels [65]. When HPH is inactivated, HIF levels rise and transcription of the downstream genes occurs (Figure 4). There is presently no known sporadic counterpart to HLRCC renal malignancy and no evidence to support a relationship between the FH mutation and tumorigenesis in nonfamilial cancers. However, in 93% of HLRCC families (52/56), germline alterations were identified in the *FH* gene [66]. 

Potential areas of systematic therapy for HLRCC will likely be designed to prevent increased HIF levels or target the transcription products of VHL-independent HIF accumulation, such as VEGF and TGF-*α*/EGFR. One attempt to block the downstream affects of *FH* inactivation is through the use of erlotinib, an oral EGFR tyrosine kinase inhibitor (TKI). A multicenter phase II trial of this agent in patients with locally advanced and metastatic papillary RCC reported an overall RECIST response rate of 11% with an additional 24 patients (53%) experiencing stable disease [67].

Combination therapy with an mTOR inhibitor or VEGF pathway antagonist may potentiate the single agent activity of erlotinib. A phase II trial of erlotinib (EGFR TKI) in combination with bevacizumab (monoclonal antibody against VEGF) is currently underway and is one of the trials designed to evaluate this strategy [68].

## 5. Birt-Hogg-Dubé

Birt-Hogg-Dubé (BHD) is a hereditary renal cancer syndrome that is associated with chromophobe renal tumors. The inheritance pattern is autosomal dominant, and affected individuals develop cutaneous fibrofolliculomas, pulmonary cysts, spontaneous pneumothoraces, and renal tumors [70]. In identified genetic carriers, renal tumors were observed in 14–34%, spontaneous pneumothoracies in 23%, and pulmonary cysts in 83% [14]. The majority of the renal tumors are of chromophobe histology (33%), hybrid tumors (50%), and oncocytomas (5%). Multifocal oncocytosis is seen in the surrounding renal parenchyma in 50% of the affected individuals. In addition, clear cell RCC is seen in patients with BHD [71]. 

The BHD gene, folliculin (*FLCN*), was localized to chromosome 17 and was subsequently identified at 17p11.2 [72, 73]. *FLCN* is altered as a result of insertions, deletions, or nonsense mutations [74]. *FLCN* deficiency activates the mammalian target of rapamycin (mTOR) pathway [75]. The *FLCN* gene has the traits of a tumor suppressor and requires two mutations with the second hit inactivating the gene [76].

**Figure 5:**
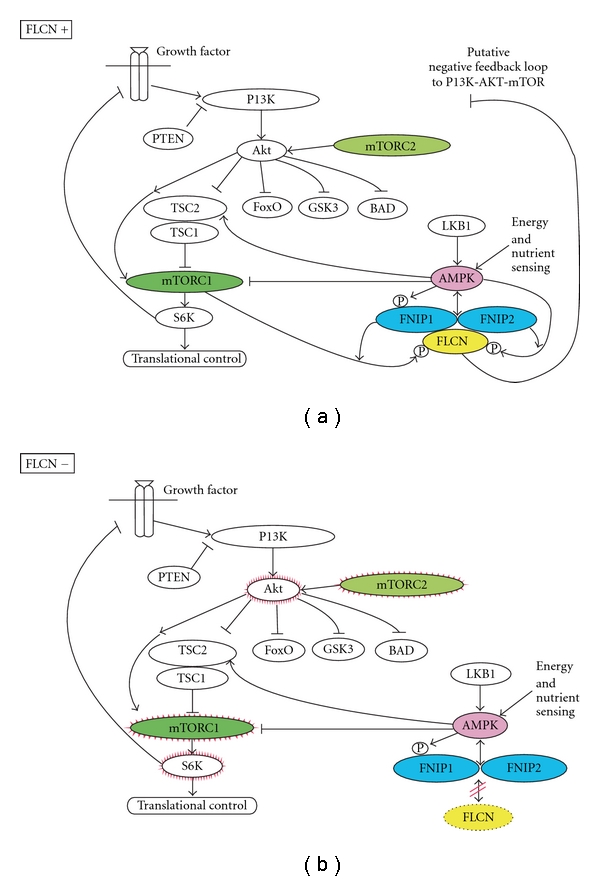
FLCN pathway. (a) *FLCN* is the gene associated with BHD. Normal FCLN protein complexes with FNIP1, FNIP2, and AMPK. This complex is phosphorylated by a rapamycin-sensitive kinase (mTORC1). (b) When FLCN is altered, it fails to complex and allows activation of AKT, mTORC1, and mTORC2. (From Hasumi et al. [69], with permission.)

*FLCN* forms a complex with folliculin-interacting proteins (FNIP1 and FNIP2). These components intern bind to AMP-activated protein kinase (AMPK). AMPK acts to sense cellular energy and assists in the regulation of the mTOR activity level [75, 77]. In tumors that are noted to have *FLCN* alterations in both alleles, mTOR activation (mTORC1 and mTORC2) has been observed [69]. Rapamycin inhibits the mTOR pathway and has been noted to prolong survival and reduce renal manifestations of BHD in *FLCN* knockout mice [78]. This represents a potential therapeutic role for mTOR inhibitors in patients affected by BHD-related renal tumors. At present, the role of mTOR pathways in sporadic chromophobe tumors is under investigation (Figure 5).

## 6. Surgical Management

Patients with FRC are likely to develop multifocal, bilateral, and recurrent renal tumors. In managing these patients, two goals are paramount: prevention of metastatic disease and preservation of renal function. At the National Cancer Institute, the first goal has been achieved largely through diligent surveillance serial cross-sectional imaging and observation until the dominant lesion achieves a size of 3 cm [79]. When a lesion becomes 3 cm, surgical intervention is recommended. In patients adhering to this “3 cm rule”, none developed metastatic disease with more than 10 years followup [80]. It should be noted, however, that the 3 cm rule was initially developed in the VHL population and later expanded to include HPRC and BHD patients. Patients with HLRCC and *any* evidence of solid tumor are offered surgical extirpation given the highly aggressive nature of their disease. Active surveillance is not recommended for HLRCC patients with renal tumors. The second goal of renal preservation has been achieved with a committed approach to nephron-sparing surgery (NSS) using open, laparoscopic, and robotic approaches [81–83]. 

The development of locally recurrent renal tumors is rare in sporadic disease but more common in patients with FRC, although it is difficult to distinguish recurrent disease from adjacent *de novo* tumor in the majority of cases. Disease recurrence in the ipsilateral renal unit has been described at the same site or elsewhere in the kidney after both partial nephrectomy and ablative therapy (cryoablation or radio frequency ablation) [84–86]. This scenario presents a complicated management dilemma, and this particular situation is occurring with greater frequency as a result of the increased use of nephron sparing surgery [82]. In the setting of local recurrence, management options include observation, initial or repeat ablation, repeat or salvage partial nephrectomy, radical nephrectomy, or systemic therapy. Each management option presents a unique array of risks and benefits. The majority of renal units can be salvaged in the face of disease recurrence. However, it comes at the cost of increased perioperative complication rates [82].

Reoperation for locally recurrent disease is often associated with a difficult dissection due to disruption of normal anatomic tissue planes as well as perinephric scarring. Complication rates increase with the number and complexity of repeated interventions on the ipsilateral kidney. Greater operative times, blood loss, and perioperative complications are typically observed [87]. The overall major complication rate approaches 20% with repeat partial nephrectomy [88], whereas complication rates in surgically naïve patients range from 11% to 13% [89, 90]. The most common complication among reoperative patients is urinary leak. However, this was noted to resolve in all patients [88]. Other complications include bowel injury, renovascular injury, and, rarely, loss of a renal unit or death. Although the risks associated with repeated surgical intervention on the same renal unit are significant, they are offset by the benefits of avoiding the morbidity and mortality associated with renal replacement therapy. While the treatment algorithm offers many branch points, the complexity and increased risk associated with these procedures demand referral to an experienced surgeon at a medical center that can provide comprehensive care.

In cases of metastatic disease, surgical treatment options alone are typically inadequate. Systemic therapies that target VEGF, VEGFR, and mTOR are commonly used. While the agents in these drug classes have been shown to improve progression-free survival and overall survival, durable complete responses are uncommon. For patients with clear cell RCC and a good performance status, treatment with IL-2 is often considered as it is the only systemic therapy shown to provide the opportunity for durable complete responses [91]. Given the improving yet limited efficacy of systemic therapy for RCC, aggressive surgical extirpation, including lymphadenectomy and metastectomy in select patients, should remain the treatment of choice whenever technically feasible [92, 93].

## 7. Conclusions

FRC is a heterogeneous disease and represents a spectrum of cancer gene pathways. Having a high suspicion for the presence of FRC allows for the appropriate counseling, screening, and surveillance of the patient and other potentially affected kindred. The complexity of these pathways requires unique therapeutic management strategies for each cancer syndrome. Understanding these pathways has led to improved management of affected patients. Using a strategy aimed at renal preservation and prevention of systemic disease, the care of FRC patients and families has been optimized. However, it is clear that no single strategy offers a comprehensive solution. The continuation of translational research, diligent surveillance programs, NSS, and the management of systemic disease with immunotherapy and novel-targeted therapies appears to be the most efficacious contemporary strategy.

## Figures and Tables

**Table 1:** Familial renal cancer syndromes.

Syndrome	Phenotype	Renal cancer manifestation	Gene	Chromosome	Mendelian Inheritance in Man (MIM) number
Von Hippel-Lindau (VHL)	Renal tumors, adrenal pheochromocytomas, retinal angiomas, central nervous system hemangioblastomas, pancreatic cysts and neuroendocrine tumors, endolymphatic sac tumors, epididymal and broad ligament cystadenomas	Clear cell renal cell carcinoma	*VHL*	3p25	193300

Hereditary papillary renal carcinoma (HPRC)	Bilateral, multifocal renal tumors	Papillary renal cell carcinoma type 1	*MET*	7q31	164860

Hereditary leiomyomatosis and renal cell carcinoma (HLRCC)	Skin and uterine leiomyomas, renal tumors	Papillary renal cell carcinoma type 2	*FH*	1q42-43	605839

Birt-Hogg-Dubé (BHD)	Cutaneous fibrofolliculomas, lung cysts, spontaneous pneumothorax, renal tumors	Hybrid oncocytic, chromophobe, and clear cell renal cell carcinoma; oncocytoma	*FLCN*	17p11	135150
